# Dexamethasone Stimulation Test in the Diagnostic Work-Up of Growth Hormone Deficiency in Childhood: Clinical Value and Comparison With Insulin-Induced Hypoglycemia

**DOI:** 10.3389/fendo.2020.599302

**Published:** 2020-12-09

**Authors:** Alessandro Cattoni, Silvia Molinari, Francesco Medici, Paola De Lorenzo, Maria Grazia Valsecchi, Nicoletta Masera, Marta Adavastro, Andrea Biondi

**Affiliations:** ^1^Department of Pediatrics, Università degli studi di Milano-Bicocca, Fondazione MBBM, San Gerardo Hospital, Monza, Italy; ^2^Center of Biostatistics for Clinical Epidemiology, School of Medicine and Surgery, Università degli studi di Milano-Bicocca, Monza, Italy; ^3^Tettamanti Research Center, Pediatric Clinic, Università degli Studi di Milano-Bicocca, Monza, Italy

**Keywords:** GH deficiency, dexamethasone stimulation test, insulin tolerance test, arginine stimulation test, human growth hormone

## Abstract

**Context:**

dexamethasone has been demonstrated to elicit GH secretion in adults, but few data are available about its effectiveness as a provocative *stimulus* in the diagnostic work-up of GH deficiency (GHD) in childhood.

**Objective:**

to assess the clinical value of dexamethasone stimulation test (DST) as a diagnostic tool for pediatric GHD.

**Design and setting:**

retrospective single-center analysis. The study population included 166 patients with a pathological response to arginine stimulation test (AST, first-line test) and subsequently tested with either insulin tolerance test (ITT) or DST as a second-line investigation between 2008 and 2019.

**Main outcome measures:**

comparison between GH peaks and secretory curves induced by ITT and DST; degree of agreement between DST and AST *versus* ITT and AST.

**Results:**

the pathological response to AST (GH peak < 8 ng/mL) was confirmed by an ITT in 80.2% (89/111) of patients and by a DST in 76.4% (42/55), with no statistical difference between the two groups (*p* value 0.69). Mean GH peaks achieved after ITT and DST were entirely comparable (6.59 ± 3.59 *versus* 6.50 ± 4.09 ng/ml, respectively, *p* 0.97) and statistically higher than those elicited by arginine (*p* < 0.01 for both), irrespectively of the average GH peaks recorded for each patient (Bland-Altman method). Dexamethasone elicited a longer lasting and later secretory response than AST and ITT. No side effects were recorded after DST.

**Conclusions:**

DST and ITT confirmed GHD in a superimposable percentage of patients with a pathological first-line test. DST and ITT share a similar secretagogue potency, overall greater than AST.

## Introduction

Growth hormone deficiency (GHD) is a treatable cause of short stature in children. Although treatment with growth hormone (GH) has been available for more than half a century, the diagnostic process is still challenging due to the lack of a standardized reliable tool for a certain diagnosis of GHD (1).

The diagnosis is mostly clinical, based on auxological features. Nevertheless, once alternative etiologies of short stature and reduced growth velocity have been ruled out, an insufficient GH response to stimulating tests is mandatory to diagnose GHD in childhood (2, 3).

The validity and reproducibility of GH testing is controversial, owing to GH variability depending on pubertal stage, age and body mass index, the lack of reference ranges for GH values in normally growing children, and the use of different laboratory assays for measuring GH values. In addition, as the diagnostic threshold to distinguish between GH-deficient and non-deficient children after pharmacological stimulation is arbitrary, it remarkably varies among different countries, and it is often established irrespectively of the specific stimulus administered (4, 5). In Italy, the cutoff for stimulated GH peaks was set at 10 ng/ml until 2014 and thenceforth reduced to 8 ng/ml (6, 7).

In addition, there is no consensus on the best provocative stimuli to assess GH deficiency and predict successful treatment with recombinant human GH. The most commonly used secretagogues include insulin-induced hypoglycemia [insulin tolerance test (ITT)], arginine [arginine stimulation test (AST)], clonidine, glucagon, levodopa, and growth hormone releasing hormone (GHRH) (4, 8, 9).

Most of these investigations may expose children to potential risks and side effects, i.e. severe hypoglycemia, vomiting or hypotension. In particular, though commonly regarded as the diagnostic gold standard for GHD in the transitional period and in adulthood, ITT is contraindicated in very young children or in case of coexistent non-endocrine disorders, such as history of seizures, coronary disease, and inborn error of metabolism involving abnormal glucose homeostasis due to the risks related to severe and persistent hypoglycemia (2, 10–13). Accordingly, the scientific community has historically searched for safer but accurate dynamic tests for children, especially for those with additional clinical fragilities.

Since 1970, many studies have been reporting that acute administration of either oral or intravenous dexamethasone in adults induced GH secretion through GH gene transcription stimulation and mRNA synthesis, somatostatin inhibition and improved pituitary sensibility to GHRH (14–20).

Conversely, very little is known about the effectiveness and diagnostic accuracy of dexamethasone in eliciting a GH secreting response in childhood. As a consequence, current pediatric guidelines (2) do not routinely include dexamethasone among GH-secretagogues agents commonly used in pediatrics, and its clinical use is still very limited and restricted to patients with concomitant clinical conditions contraindicating other dynamic tests. Nevertheless, its safe clinical profile makes dexamethasone an appealing option among pharmacological stimuli.

To the best of our knowledge, only three studies, held on small cohorts of pediatric patients, have reported the potential diagnostic reliability of dexamethasone stimulation test (DST) (21–23).

The primary aim of the present analysis was to assess the clinical value of dexamethasone stimulation test as a diagnostic tool in GHD in a wide pediatric population. To achieve this purpose, we compared two cohorts of children who underwent either ITT or DST as a second-line GH provocation test after a pathological response to arginine and analyzed the degree of agreement between GH peaks achieved after ITT or DST and those recorded after AST. In addition, we aimed at assessing GH peaks, GH curves, and secretory potency recorded after AST, ITT, or DST.

## Materials and Methods

We performed a retrospective observational single-center study. The overall cohort consisted of all non-syndromic pediatric patients with suspected GHD based on clinical and auxological criteria (6, 7) who presented with a pathological response after AST (first-line test) and who were subsequently tested with either ITT or DST as a second-line test between 1^st^ January 2008 and 31^st^ December 2019 at the pediatric endocrinological service of San Gerardo Hospital, Monza, Italy. All the patients enrolled were younger than 16 years at the time of GH-IGF-I axis assessment.

Conditions that led to patients’ exclusion were: annual linear growth velocity <2 cm per year in post-pubertal patients, clinical, or laboratory data suggestive of hepatic or renal impairment at the moment of GH-IGF-I assessment, syndromic conditions related to GHD and/or poorly controlled pituitary insufficiencies of axes other than GH.

Among the second-line tests prescribed, ITT was deemed as the first choice. Patients were tested with DST only if they presented with one or more of the following criteria: body weight < 10 kg, age < 3 years, reported episodes of unexplained symptomatic hypoglycemia, diagnosis of inborn errors of metabolism involving risk for severe hypoglycemia and/or a previous medical history consistent with cardiac or epileptic disorders.

In order to assess the performance of the second-line tests, the study population was identified within the overall cohort by additionally excluding those patients who had achieved a normal GH peak during a confirmatory test (ITT or DST) at time points t-20 min or at time 0 (t0). This is motivated by the fact that a normal GH peak detected prior to drug administration would not depend on the kind of pharmacological agent administered and it could not contribute in defining the effectiveness of the *stimulus* in eliciting a response. Conversely, patients with a pathological GH peak (<8 ng/ml) measured prior to the administration of the *stimulus* (at t-20/-30 or t0) were included, as the pharmacological agent could not elicit an adequate GH response, confirming the positive result of the test.

Finally, in order to compare GH peaks elicited by different dynamic tests (secondary endpoint), we specifically selected a subgroup of patients who presented with a stimulated GH peak after t0 at both first- and second-line stimulation tests [Group GHPAT0 (GH Peak After Time 0)].

With reference to the second-line testing undertaken (ITT or DST), all the patients enrolled were classified into the following sub-cohorts: GH_ITT_ and GH_DST_, respectively.

Informed consent was obtained from parents or legal guardians.

### Definitions and Infusion Protocols 

A dynamic test was classified as pathologic when the peak value elicited was lower than 8 ng/mL (Italian Medicines Agency Consensus Guidelines) (6). In a patient with consistent clinical and auxological data, the diagnosis of GH deficiency was confirmed by the finding of a pathological GH peak value at two standardized provocation tests, performed in two different days and with a diverse secretagogue agent.

Dynamic tests were performed according to the following infusion protocols:

- AST: intravenous arginine at the dose of 0.5 g/kg was infused in 30 min. Blood samples for GH were collected at the following time points: -30, 0, 30, 45, 60, 90, and 120 min after arginine administration.
- ITT: intravenous regular insulin at the dose of 0,1 U/kg was infused. Blood samples for GH were collected at the following time points: -20, 0, +15, +30, +45, +60, +90, and + 120 after insulin administration. The test was considered valid in the presence of symptoms consistent with hypoglycemia and/or a reduction of glycemia of 50% or more and/or a glycemia < 45 mg/dl, recorded at least at one timepoint.
- DST: intravenous dexamethasone at the dose of 2 mg/sm was infused. Blood samples for GH were collected at the following time points: 0, +60, +90, +120, +135, +150, + 165, +180, +195, +210, +225, and +240 after dexamethasone administration. Patients underwent DST as they presented with contraindications to the infusion of insulin, glucagon and clonidine.

All investigations were performed with fasted patients. Sexual steroids were administered as a priming prior to the test in prepubertal patients with a bone age >10 years. In boys, a single dose of 100 mg of *depot* testosterone was administered intramuscularly 7 days prior to the dynamic test. In girls, 20 micrograms of ethinylestradiol was administered once a day for three days before testing.

### Data Collection and Analysis 

We recorded demographic, anthropometric, biochemical and radiological data. In order to compare children of different ages and sex, anthropometric data (height, weight and body mass index) were expressed as standard deviation scores (SDS) according to World Health Organization (WHO) reference centiles. Standing height was measured with a Harpenden stadiometer.

Both IGF-I and GH were assayed with a fully automated chemiluminescence analyzer (LIASON XL, produced by DiaSorin S.p.A).

IGF-I values were normalized according to patient’s age and expressed as IGF-I SDS according to the assay-specific reference ranges provided by DiaSorin S.p.A (24, 25).

Bone age was assessed *via* Greulich and Pyle method (26); in case of uncertainty, Tanner & Whitehouse method (27) was applied.

Mean and standard deviation (SD) values, interquartile ranges and median were used to describe the study population. To compare categorical variables, Fisher exact test and Chi-square test were used, whereas continuous variables were compared with Wilcoxon rank-sum test. Bonferroni correction was used in case of multiple comparisons. The Bland-Altman approach was used to analyze the agreement between the different tests. The differences between the two log-transformed measures on each subject were plotted against their average value. After excluding any dependence, the 95% range for the difference, calculated from twice the standard deviation and the hypothesis of zero mean difference (bias), was examined with a paired t-test. Multiple regression models were used to assess the potential impact of demographical and auxological factors on GH peaks achieved during the second-line tests and a logistic model was applied to compare the likelihood of receiving a diagnosis of GHD (expressed in terms of odds ratio, OR) by confirmatory test, adjusting for demographical and auxological factors. All tests were two-sided. Statistical analysis was performed using SPSS version 24 statistical package (SPSS IBM, New York, USA) and SAS 9.4.

## Results

### Overall Cohort and Study Population

One hundred ninety-nine otherwise healthy patients aged 1.01 to 15.73 years (mean 8.24 ± 3.35) sequentially underwent AST (first-line test) and either ITT or DST (second-line test) at our Center between 1^st^ January 2008 and 31^st^ December 2019. One-hundred-fourteen (57.3%) were males.

From an auxological perspective, mean baseline height SDS was -2.45 ± 0.88. Amongst 147/199 (73.9%) patients whose height SDS was below -2 SDS, 38 (25.9%) presented with severe short stature (recorded height below < -3 SDS). The remaining 52/199 patients (26.1%) whose height was above -2 SDS were tested because they presented with either a severe impairment in height velocity recorded at least 6 months apart or a mild height velocity decrease in patients remarkably shorter than the expected midparental target potential (*Δ target-height SDS* > 1.5). Overall, the target height potential was -0.58 ± 0.92 SDS, while *Δ target-height SDS* was 1.85 ± 1.07.

**Figure 1:**
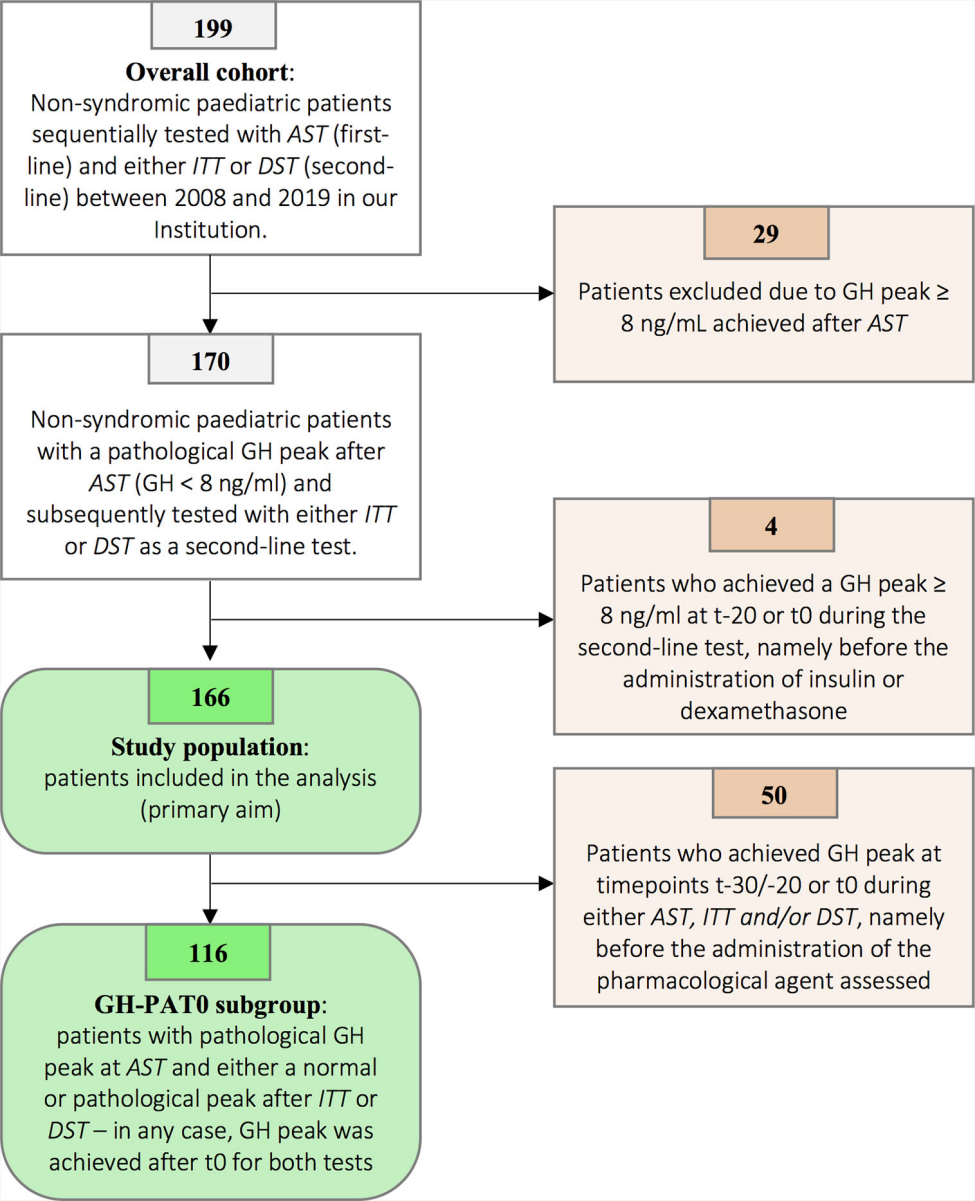
Patients’ selection flowchart. Among 199 patients initially tested with *AST* and subsequently with either *ITT* or *DST*, 29 presented with a peak value achieved after *AST* ranging from 8.00 to 9.99 ng/ml. According to the previous versions of the Italian national guidelines, GH peaks below 10.00 ng/mL were recorded as pathological and patients therefore underwent a second confirmatory stimulation test. In order to harmonize the result according to the latest versions of the Italian guidelines (normal GH peak ≥ 8.00 ng/ml), 29 patients with GH peaks ≥ 8.00 ng/ml were excluded. Four out of the remaining 170 patients have been excluded as a normal GH peak at second-line test was achieved at t-20 or t0. As a consequence, the diagnostic role of either *ITT* or *DST* could not be assessed, as the peak (classified as normal) was irrespective of the kind of pharmacological agent administered. Overall, 166 patients were included in the analysis and represented the study population. Finally, in order to specifically assess the direct effect of insulin and dexamethasone on GH secretion (secondary endpoints), we identified 116 patients to be included in the GHPAT-0 (GH Peak After Time 0) subgroup. Indeed, in 50 amongst the 166 patients included in the study the GH peak was achieved before the administration of the pharmacological agent and thus did not allow to assess the differential contribution of insulin and dexamethasone on pituitary secretion.

According to the enrollment criteria listed above, the stepwise selection exposed in **Figure 1** led to the identification of a study population of 166 patients out of 199 patients of the overall cohort (see Figure caption for additional details).

### Pathological Response After ITT and DST

One hundred eleven out of the 166 patients (66.9%) included in the study population sequentially underwent AST and ITT (GH_ITT_ subgroup), while children tested with DST as a confirmatory test were 55/166 (33.1%, GH_DST_ subgroup). A comparison of the auxological and demographic features of the patients included in these subgroups is reported in **Table 1**. Overall, age at the time of baseline auxological assessment, height SDS and *Δ target-height SDS* were statistically different between the two groups, with patients from the GH_DST_ group being younger and shorter that those from GH_ITT_.

**Table 1:** Auxological and demographic features of patients sequentially tested with AST and ITT (GH_ITT_), and with AST and DST (GH_DST_).

	GH_ITT_	GH_DST_	*p value*
**Number of patients**	111 (66.9%)	55 (33.1%)	
**Gender (males)**	M 69 (62.2%)	M 30 (54.5%)	0.40
**Age (years)**	9.23 ± 3.12	6.13 ± 3.13	<0.0001
**Height SDS**	-2.26 ± 0.57	-2.85 ± 1.28	<0.0001
**BMI SDS**	-0.62 ± 1.14	-0.22 ± 1.47	0.15
**Δ CA – BA (years)**	1.82 ± 1.32	1.85 ± 1.02	0.67
**Δ target-height SDS**	1.62 ± 0.87	2.4 ± 1.38	<0.0001
**IGF-I SDS**	-1.31 ± 0.92	-1.43 ± 1.26	0.09

CA, chronological age; BA, bone age; ITT, insulin tolerance test; DST, dexamethasone stimulation test.

Eighty-nine out of 111 patients (80.2%) from the GH_ITT_ group were diagnosed with GH deficiency, as ITT confirmed the pathological finding of the AST previously performed. On the other hand, DST confirmed the pathological outcome of AST in 42 out of 55 (76.4%) children from the GH_DST_ group. These data clearly highlight that the proportion of patients ultimately diagnosed with GH deficiency did not depend on the second-line test adopted. Indeed, ITT and DST provided pathological responses, in agreement with the results previously collected with an AST, in a comparable percentage of patients (80.2% in ITT versus 76.4% in DST; *p value* 0.69, **Table 2**).

**Table 2:** Distribution of patients enrolled according to the results of ITT and DST test: GH deficiency confirmed if GH peak achieved after ITT or DST < 8 ng/ml. GH excluded if GH peak ≥ 8 ng/ml.

	GH deficiency excluded by second test	GH deficiency confirmed by second test	Total
**GH_ITT_**			
*Number of patients*	22	89	111
*% among patients* * tested with ITT*	19.8%	80.2%	100%
* % among the total* * number of patients * * enrolled*	13.3%	53.6%	66.9%
**GH_DST_**			
* Number of patients*	13	42	55
* % among patients * * tested with DST*	23.6%	76.4%	100%
*% among the total * * number of patients * * enrolled*	7.8%	25.3%	33.1%
**Total**			
* Number of patients*	35	131	166
* % among the total * * number of patients enrolled*	21.1%	78.9%	100%

Overall, 131 out of 166 (78.9%) patients were diagnosed with GHD and therefore underwent a diagnostic brain MRI. The imaging showed findings consistent with a normal appearance of the hypothalamic-pituitary area (HPA) in 109 children (83.2%), pituitary hypoplasia in 17 cases (13.0%), empty sella in 3 (2.3%) and a neoplasm involving the HPA in 2 patients (1.5%). **Table 3** provides radiological findings, hormonal profiles and auxological features of the 131 patients diagnosed with GH deficiency within the study population.

**Table 3:** Radiological findings, pituitary hormonal profile and auxological features of the 131 patients diagnosed with GH deficiency within the study population.

Second-line test	MRI findings		Associated pituitary hormonal deficiency		Auxological presentation				
	Finding	Number of patients (%)	Multiple hormonal deficiency	Isolated GH deficiency	Height SDS <-3	Height SDS < -2 and height velocity < -1 SDS	Height velocity SDS < -2	Height SDS < -2 and Δ target-height SDS > 2	Height SDS > -2 and Δ target-height SDS > 2
**DST**	Normal pituitary	32/42 (76.2%)	0/32 (0%)	32/32 (100%)	12/32 (37.5%)	6/32 (18.8%)	0/32 (0%)	12/32 (37.5%)	2/32 (6.2%)
	Pituitary hypoplasia	7/42 (16.6%)	1/7 (14.3%)	6/7 (85.7%)	3/7 (42.8%)	2/7 (28.6%)	1/7 (14.3%)	1/7 (14.3%)	0/7 (0%)
	Empty sella syndrome	2/42 (4.8%)	0/2 (0%)	2/2 (100%)	1/2 (50.0%)	0/2 (0%)	0/2 (0%)	1/2 (50.0%)	0/2 (0%)
	Neoplasm involving the pituitary	1/42 (2.4%)	1/1 (100%)	0/1 (0%)	0/1 (0%)	0/1 (0%)	1/1 (100%)	0/1 (0%)	0/1 (0%)
	Total	42	2/42 (4.8%)	40/42 (95.2%)	16/42 (38.1%)	8/42 (19.0%)	2/42 (4.8%)	14/42 (33.3%)	2/42 (4.8%)
**ITT**	Normal pituitary	77/89 (86.6%)	0/77 (0%)	77/77 (100%)	7/77 (9.1%)	23/77 (29.9%)	5/77 (6.5%)	28/77 (36.4%)	14/77 (18.2%)
	Pituitary hypoplasia	10/89 (11.2%)	0/10 (0%)	10/10 (100%)	1/10 (10%)	3/10 (30%)	3/10 (30%)	3/10 (30%)	0/10 (0%)
	Empty sella syndrome	1/89 (1.1%)	0/1 (0%)	1/1 (100%)	1/1 (100%)	0/1 (0%)	0/1 (0%)	0/1 (0%)	0/1 (0%)
	Neoplasm involving the pituitary	1/89 (1.1%)	1/1 (100%)	0/1 (0%)	0/1 (0%)	0/1 (0%)	1/1 (100%)	0/1 (0%)	0/1 (0%)
	Total	89	1/89 (1,1%)	88/89 (98.9%)	8/89 (9.0%)	23/89 (25.8%)	13/89 (14.6%)	31/89 (34.9%)	14/89 (15.7%)

### Comparison Between Insulin- and Dexamethasone-Induced GH Peaks

In 50 out of 166 patients enrolled, a pathological peak was recorded at t-30/-20 or at t0 during either AST, ITT, or DST. Accordingly, we included the remaining 116/166 children in the GH-PAT0 subgroup, specifically designed in order to assess and compare GH peaks directly elicited by either insulin or dexamethasone (see Materials and Methods section).

**Table 4** describes the mean and median GH peaks achieved among the 73/116 patients tested with ITT as a second-line test (GH-PAT0_ITT_) and the 43/116 children tested with DST (GH-PAT0_DST_).

**Table 4:** Median and mean GH peaks achieved after *AST* and *ITT*/*DST* in the GH-PAT0_ITT_ and GH-PAT0_DST_ subgroups.

Subgroup of patients	Number of patients	GH peaks after AST		GH peaks after second-line test (ITT/DST)	
		Median(*range*)	Mean(SD)	Median(*range*)	Mean(SD)
**GH-PAT0_ITT_**	73	5.00 (0.81–7.90)	4.87 (1.85)	6.00 (1.01–24.00)	6.59 (3.59)
**GH-PAT0_DST_**	43	4.75 (0.80–7.90)	4.58 (1.74)	5.60 (0.82–19.00)	6.50 (4.09)
**GH-PAT0**	116	4.89 (0.80–7.90)	4.76 (1.81)	5.89 (0.82–24.00)	6.53 (3.71)

The mean GH peak achieved after AST was not different in the two subgroups (4.87 ± 1.85 ng/ml *versus* 4.58 ± 1.74, respectively; *p value* of 0.57). The mean value of GH peak achieved after ITT in the GH-PAT0_ITT_ subgroup was 6.59 ± 3.59 ng/ml, superimposable to that recorded after DST in GH-PAT0_DST_ patients (6.50 ± 4.09 ng/ml; *p value* of 0.97).

### ITT and DST *versus* AST: Mean Peaks and Degree of Agreement

**Figure 2:**
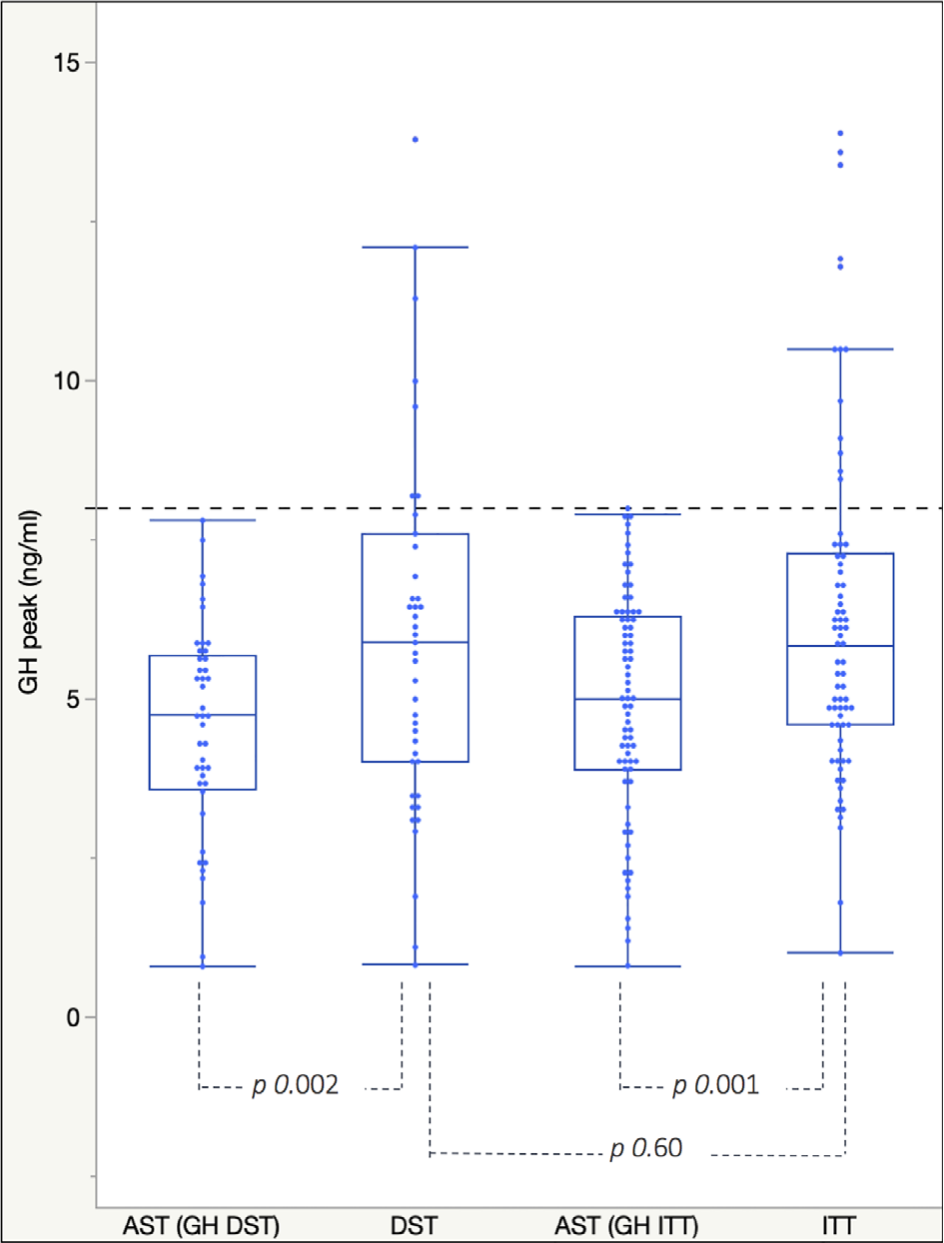
Box plots for mean and interquartile range of GH peaks achieved after *AST* and *ITT*/*DST* in the GH-PAT0_ITT_ and GH-PAT0_DST_ subgroups. The dotted blue line represents the *cut-off* of 8 ng/ml used to discriminate between GH deficient and not-deficient patients.

Mean GH peaks achieved after second-line tests were higher than those elicited by AST, both after ITT (*p value <* 0.001) and after DST (*p value* of 0.0016, **Figure 2**).

Overall, GH peaks elicited by insulin-induced hypoglycemia and dexamethasone were extremely similar to each other and higher than those recorded in patients previously tested with AST.

**Figure 3:**
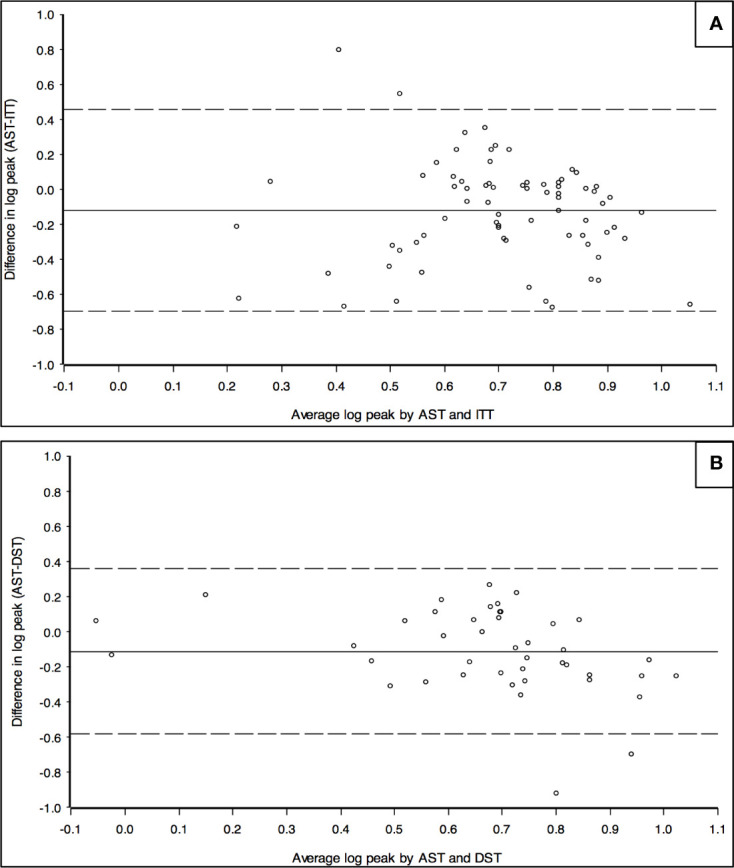
Bland-Altman chart to assess the agreement between GH peaks recorded after *AST* and *ITT* **(A)** and between *AST* and *DST* **(B)**. Y axis: logarithm of the difference between the peaks of GH recorded in the same patient after *AST* and *ITT* **(A)** or between *AST* and *DST* **(B)**. X axis: logarithm of the mean value of GH peaks recorded in the same patient after AST and ITT **(A)** or after *AST* and *DST* **(B)**. The chart represents the mean value of the difference between the two results recorded in each patient (solid horizontal line) and it is statistically lower than 0 in both Figures. Dotted horizontal line represent -2 and +2 SD.

In order to investigate the agreement between AST and ITT and AST and DST, the recorded GH peaks were plotted according to the Bland-Altman approach. As showed in **Figure 3**, the difference between the two paired measurements (i.e. difference between GH peaks achieved after AST and after a second-line test in the same patient, Y axis) is plotted against the mean of the two measurements (i.e. the mean of the peaks recorded at test 1 and test 2, X axis). The estimated mean differences (after logarithm transformation) of GH peaks after AST *versus* ITT were significantly lower than zero, thus meaning that GH peaks after AST tended to be lower than after ITT (estimated mean difference was -0.12, SD = 0.29, *p value* < 0.001). A similar finding was recorded when GH peaks after AST were compared with those after DST (estimated mean difference was -0.11, SD = 0.25; *p value* of 0.004). The difference between AST and either ITT or DST was consistent for all the progressively increasing mean GH peaks plotted along the X axis, as demonstrated by the limited scattering of the values recorded, essentially included between +2 and -2 SD of the mean value (solid horizontal line). In conclusion, both DST and ITT provided GH peaks statistically higher than AST, irrespectively of the severity of GH deficiency diagnosed.

### Point-By-Point Comparison of the Secretive Curves Recorded After AST, ITT, and DST

**Figure 4:**
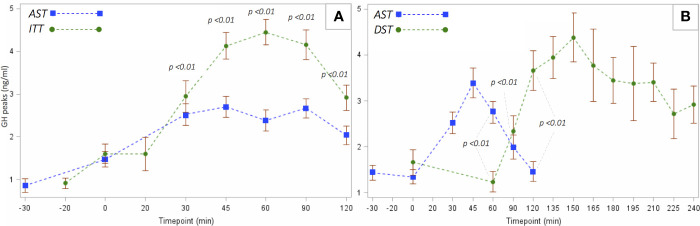
Comparison of the mean GH values recorded at each timepoint between *AST* and *ITT* **(A)** and between *AST* and *DST* **(B)**. Bonferroni-corrected α: 0.01 for GH_ITT_ and 0.017 for GH_DST_.

**Figure 4** shows the GH mean secretory curves induced in patients tested with AST and ITT (panel A) and with AST and DST (panel B).

By comparing AST and ITT (**Figure 4A**), the secretion curves show that GH mean values were statistically higher (*p value* < 0.01, Bonferroni-corrected α: 0.01) after insulin-induced hypoglycemia all throughout the sample collection, from t30 onwards, till the end of the test. Within the GH_ITT_ group, GH peak was achieved more frequently at t45 during AST and at t60 at ITT.

On the other hand, a point-by-point comparison between AST and DST can be performed only partially (**Figure 4B**), as the duration of the latter is remarkably longer. Also within the GH_DST_ subgroup, GH peaks were more frequent at t45 after AST (consistently with the GH_ITT_ cohort) and at t150 after the administration of dexamethasone.

### Factors Affecting GH Peaks and GH Response

Age-adjusted IGF-I SDS levels (classified as <-2 SDS, -2 SDS ≤value≤-1 SDS, > -1 SDS) showed no statistically significant impact on GH response achieved after either ITT (*p value* of 0.48 at Fisher’s exact test) or DST (*p value* of 0.62).

In order to jointly assess the potential impact of demographic, auxological and biochemical factors on the peak achieved after different GH stimulation tests, we applied a multiple regression model in the GH-PAT0_ITT_ and in the GH-PAT0_DST_ subgroups, separately. In the DST cohort, none among sex, age, height SDS, *Δ target-height SDS* and age-adjusted IGF-I SDS significantly affected GH peaks. On the other hand, BMI-SDS showed a statistically significant negative impact on GH secretion, with GH peaks presenting with a drop of about 6% as a result of 1 SDS increase in BMI (*p value* of 0.04).

With ITT, we found a statistically significant impact for gender, stature and BMI-SDS. In detail, peaks were statistically lower in female patients (*p value* of 0.04), with mean GH values being 11% lower than males, after ITT. In addition, shorter patients presented with lower GH peaks (*p value* of 0.03), with a drop of about 11% of the peak as a result of a 1 SDS reduction in height. Furthermore, GH peaks showed a 5% drop as a result of 1 SDS increase in BMI (*p value* of 0.04).

Finally, the performance of the two second-line tests in terms of the final outcome (i.e. pathological *versus* normal response) was assessed with a logistic model on the cohort of 116 patients from the GH-PAT0 group adjusting for the abovementioned covariates. No statistically significant difference between DST and ITT was observed (OR 1.73, 95% C.I. 0.46- 6.52, *p value* of 0.42).

## Discussion

Growth hormone stimulation tests are key elements of the diagnostic work-up of GH deficiency in childhood. These investigations are time-consuming, expensive and require active surveillance by a dedicated team of healthcare personnel due to the clinical risks that they potentially involve.

In detail, concerns have been raised during the last decades about the hazards of ITT (13), historically regarded as the diagnostic *gold standard*. Though its overall safety in tertiary care Centers has been more recently demonstrated (28), the test implies a remarkable distress in younger and lower-weight children and potential adverse or fatal events related to pharmacologically-induced symptomatic hypoglycemia in patients with underlying disorders that contraindicate the procedure, such as coronary disease, inborn error of metabolism involving hypoglycemia and epilepsy. In addition, symptomatic hypokalemia potentially leading to cardiac arrhythmias should be listed among the adverse events of ITT, though less frequently described (29).

With few exceptions, most of the alternative dynamic tests available may anyway expose fragile children to the risk of developing side effects (i.e. hypotension after clonidine, late-onset hypoglycemia after glucagon). Arginine and dexamethasone stimulation tests present with the best safety profile also in selected categories of children. While the first is widely used world-wide, few data are available about the effectiveness and diagnostic accuracy of dexamethasone in eliciting a GH secreting response in childhood. Accordingly, its use is still very limited and restricted to patients with concomitant clinical conditions contraindicating other dynamic tests.

The clinical need for safe stimulation procedures is exacerbated by the fact that in most countries two pathologic results after different dynamic tests are needed to confirm the diagnosis of GHD, according to the indications provided by the Growth Hormone Research Society (5).

Only three studies (21–23) have assessed the clinical role of DST on small cohorts of children and, in spite of an overall agreement about the reliability of the test, outcomes about the timing and levels of peaks, secretory curves and overall strength of dexamethasone in inducing GH secretion were conflicting. Onigata and colleagues tested 10 short normal and 4 GH deﬁcient children, reporting a good differentiation between the two groups (23). On the other hand, Martul and colleagues compared the results of different GH provocative tests in 8 normal and 12 GH deﬁcient children. By assessing the area under the curve induced by dexamethasone, insulin, propranolol, clonidine and GHRH, the authors concluded that the potency of dexamethasone was similar to clonidine and greater than insulin-induced hypoglycemia. Both sensitivity and specificity of DST were satisfactory, and the test was therefore regarded as suitable to be used in the diagnostic armamentaria of GH secretion disorders. However, the sample size of this study was extremely limited (21). In a wider analysis on 63 patients, Pellini and colleagues assessed the efficiency of GH secretion after dexamethasone by comparing the peaks and secretive curves elicited by DST with those recorded after clonidine stimulation test. Though DST was demonstrated to present with satisfactory sensitivity and specificity, clonidine showed a greater potency than dexamethasone in eliciting GH secretion, in contrast with the data recorded by Martul. In addition, although some of the patients enrolled underwent an ITT as a confirmatory test, no clear comparisons between hypoglycemia- and dexamethasone-induced GH secretion curves were performed (22).

In the present analysis, we aimed at comparing DST to ITT in a cohort of patients with a pathological response after a first-line test (AST).

From a methodological standpoint, the widest sample size among the studies published so far (166 patients enrolled) and the stepwise systematical selection of patients both represent the strengths of our study. In detail, when assessing the potency of a stimulation test, GH peaks recorded before the administration of the pharmacological agent may hamper the interpretation of data and provide misleading results. In addition, when GH peak was spontaneously achieved before the administration of the *stimulus*, a physiologically increased somatostatinergic tone may subsequently dampen the GH secretory response to any pharmacological agent (30). In conclusion, by excluding patients who achieved GH peaks at t-30/-20 or t0, we were able to specifically assess the secreting profile of arginine, insulin and dexamethasone. None of the cited studies clarify how the authors managed these results, which represent a remarkable percentage amongst tested patients (in our cohort: 54 out of 199 patients, 27%, were excluded due to this potential *bias*).

Patients enrolled in the GH_DST_ cohort were statistically younger than those included in the GH_ITT_ group. This is mostly due to the fact that younger patients are more frequently tested with DST, because of the risks related to hypoglycemia in children with body weight below 10 Kg. In addition, as a decrease in height SDS is physiologically expected in toddlers, only patients with an overt growth impairment are tested during the first years of life, and that explains the lower height SDS recorded in the GH_DST_ group.

In our study population, ITT and DST showed pathological results, in agreement with the peaks achieved after AST, in a comparable percentage of patients (80.2 versus 76.4%, respectively, *p value* of 0.69). In addition, a comparison between the 73 patients enrolled GH-PAT0_ITT_ and 43 from the GH-PAT0_DST_ subgroups, homogeneous in terms of GH secretion after AST, highlighted that growth hormone peaks elicited by dexamethasone were utterly similar to those recorded after ITT (6.50 ± 4.09 ng/ml *versus* 6.59 ± 3.59 ng/ml, respectively, *p value* of 0.97) and statistically higher than after AST. Additionally, the Bland-Altman approach confirmed that the demonstrated difference between GH peaks achieved after AST and second-line tests was consistent for both ITT and DST irrespectively of the severity of GH deficiency recorded. Thus, according to the results collected, dexamethasone provides a secretory response widely comparable to insulin-induced hypoglycemia in terms of induced GH peaks and overall potency of the test. According to Martul and colleagues (21), DST was regarded as the most potent stimulation tests, with its AUC being greater than all the other hypothalamic *stimuli* (ITT included). In our opinion, the estimation of the AUC provides an interesting standpoint from a physio-pathological perspective and dexamethasone actually induces a longer-standing though late stimulation to the hypothalamic-pituitary axis, resulting in persistent GH secretion and greater area under the curve. However, as national and international guidelines agree in classifying as GH-deficient those patients whose stimulated GH peak does not achieve a pre-established cut-off, a comparison of the maximum GH levels achieved after DST and ITT is pivotal from a clinical perspective.

On the other hand, we performed a systematic evaluation of the secretion curves elicited by arginine, insulin and dexamethasone and our results confirm that the latter implies a persistently stimulated GH secretion, with GH values starting to raise at t90 and mean peak values being recorded at t135-t150. In our study population, the mean peak value was recorded slightly earlier than in the analysis by Martul (195 min) and Pellini (150–165 min) (21, 22). Nevertheless, as in 13 out of 55 patients (23.6%) tested with DST the peak was achieved during the fourth hour and in 3 (5.4%) at t240, our data do not support the hypothesis of a reduction in the total duration of the test. In addition, we believe that the long duration of DST, allowing the detection of tardive GH peaks, may partially prevent the bias of a damped GH response due to a high endogenous somatostatin tone in patients who experienced a spontaneous GH peak before the administration of the pharmacological *stimulus*.

On the other hand, the number of blood samples can be reduced: for example, the raise in mean GH values tends to occur from t90 onwards and mean growth hormone levels at t60 are extremely low in our study. As in none of the patients enrolled GH peak was recorded 60 min after the administration of dexamethasone, our conclusion is that a blood sample at this timepoint could be avoided. In addition, according to the available protocols, a blood sample is withdrawn every 15 min, but the curve described tends to be flat between t180 and t210; therefore, we suggest that the number of samples in this phase may be reduced in younger children.

Furthermore, our analysis confirmed the absolute clinical safety of DST, as none among 64 patients tested presented with either side effects or symptoms ascribable to dexamethasone administration. On the other hand, among the 135 patients tested with ITT, five children presented with prolonged hypoglycemia and oral glucose was administered before the end of the test, while two patients developed a syncope and needed intravenous glucose administration. In all these cases, the children promptly recovered and neither seizures nor major *sequelae* were recorded.

Finally, multivariate analysis demonstrated that female gender is related to a statistically significant increase in the risk of being diagnosed with GH deficiency, limited to the GH_ITT_ cohort. This is mostly due to the fact that patients from the GH_ITT_ cohort presented with an age overall older than the GH_DST_ group, when pubertal delay becomes a progressively increasing cause of transient short stature. As this condition is remarkably more frequent in males, it is possible that a significant percentage of male patients tested actually presented with this physiological condition. On the other hand, from a cultural standpoint, mild short stature is socially more accepted in females and it is likely than girls were referred to our pediatric endocrinology unit when stature impairment was more severe. Indeed, height SDS was statistically lower in females (mean height SDS: -2.65 ± 0.85) than males (-2.32 ± 0.94, *p value* of 0.02). Finally, the demonstrated negative correlation between BMI-SDS and GH peaks has been already widely described (31). In our study population, GH_ITT_ and GH_DST_ cohorts were statistically homogeneous in terms of baseline BMI-SDS levels and did not differ with reference to the negative effect of BMI on the GH peaks elicited by both ITT and DST, with a 5% and 6% drop in GH peaks for 1 BMI-SDS increase, respectively. Thus, BMI did not *bias* the comparison performed between the two sub-cohorts.

The main limitations of our analysis consist of its retrospective nature and the fact that the patients enrolled did not undergo DST and ITT sequentially; accordingly, we could not perform a direct comparison of the effects of dexamethasone and hypoglycemia in the same child. Nevertheless, as GH_ITT_ and GH_DST_ cohorts were entirely homogeneous in terms of GH peaks achieved after arginine stimulation test, a comparison of the two cohorts has been regarded as methodologically proper and statistically appropriate.

Finally, despite its safer profile, an additional limitation of DST is that the test does not allow to assess the ACTH-cortisol axis, while ITT is considered as the *gold standard* also for the study of cortisol response to stress.

In conclusion, our analysis outlined the potential diagnostic value of DST in assessing GH secretion in childhood. Indeed, dexamethasone stimulation test and insulin-induced hypoglycemia confirmed GH deficiency in a superimposable percentage of patients who presented with a pathological GH peak after a first-line dynamic test. In addition, both DST and ITT provided secretory peaks statistically greater than those recorded after arginine stimulation test.

Given the safe profile of DST, we hope that our study may promote its use in the clinical practice, with particular reference to patients for whom ITT and/or other dynamic investigations are contraindicated. In addition, though the longer-lasting execution protocol and the numerous blood samples required may discourage clinicians from prescribing DST among younger children, by systematically assessing the secretory curve following dexamethasone administration, we hereby provide practical suggestion for reducing the number of blood withdrawals prescribed.

Overall, DST should be regarded as a safe and manageable investigation to assess GH secretion in children.

## Data Availability Statement 

The raw data supporting the conclusions of this article will be made available by the authors, without undue reservation.

## Ethics Statement 

Ethical review and approval was not required for the study on human participants in accordance with the local legislation and institutional requirements. Written informed consent to participate in this study was provided by the participants' legal guardian/next of kin.

## Author Contributions 

AC conceptualized and designed the study, drafted the initial manuscript, reviewed and revised the manuscript, and approved its final version. SM drafted the initial manuscript and reviewed and revised the manuscript. FM was responsible for data collection. PL and MV performed statistical analysis and reviewed the manuscript. MA, NM, and AB reviewed the manuscript for important intellectual content. All authors contributed to the article and approved the submitted version.

## Conflict of Interest

The authors declare that the research was conducted in the absence of any commercial or financial relationships that could be construed as a potential conflict of interest.
